# Supplementary material for: Companies inadvertently fund online misinformation despite consumer backlash
Source: Nature. 2024 Jun 5;630(8015):123–31. doi: 10.1038/s41586-024-07404-1 (PMC11153133; doi:10.1038/s41586-024-07404-1)
Supplement: Supplementary file 1 — This file contains Supplementary Methods (including more details about the design of the survey experiments and the method for analysing participants' open-text responses), Supplementary Tables and Figures (including descriptive statistics, additional analyses, and robustness checks). [file 41586_2024_7404_MOESM1_ESM.pdf]

nature portfolio

https://doi.org/10.1038/s41586-024-07404-1

Supplementary information

Companies inadvertently fund online misinformation despite consumer backlash

In the format provided by the authors and unedited

Supplementary Information

Contents

1 Supplementary Methods **II**
- 1.1 Design of survey experiments II
- 1.2 Analysis III
  - 1.2.1 Consumer study outcomes III
  - 1.2.2 Analysis of consumers' text responses IV
  - 1.2.3 Decision-maker study outcomes VIII

2 Supplementary Tables and Figures **IX**
- 2.1 Descriptive results IX
- 2.2 Consumer study results XI
  - 2.2.1 Summary statistics and analyses XI
  - 2.2.2 Robustness checks XV
- 2.3 Decision-maker study results XIX
  - 2.3.1 Summary statistics and analyses XIX
  - 2.3.2 Robustness checks XXII

1 Supplementary Methods

1.1 Design of survey experiments

Figure A1: Design of the consumer survey experiment.

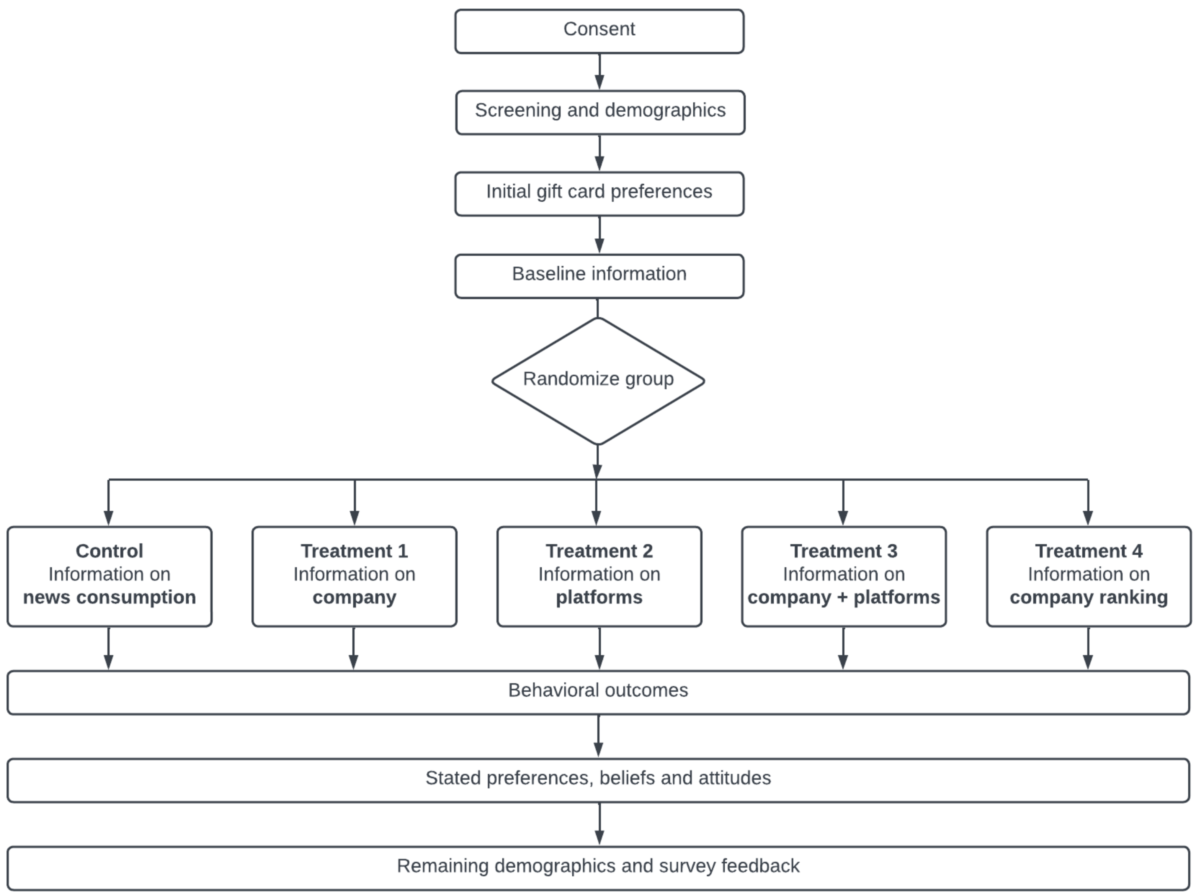

Figure A2: Design of the decision-maker survey experiment.

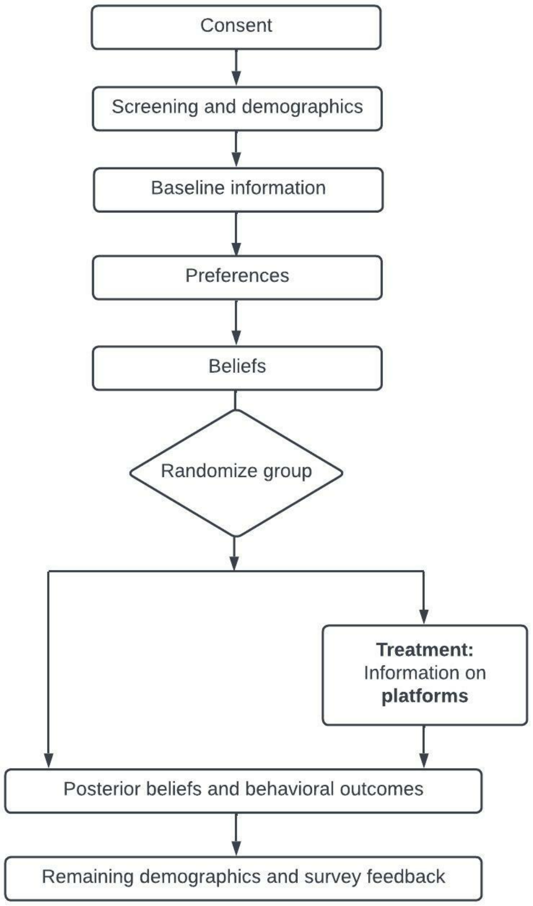

1.2 Analysis

1.2.1 Consumer study outcomes

In Supplementary Table A5, we report summary statistics for our consumer sample and demonstrate that our treatment groups are balanced across observable characteristics.

We conduct our analyses using OLS regressions to compare differences in our outcomes across treatment groups. As indicated in Tables 1 and 2, we also control for demographic and behavioral variables. The demographic controls include the respondent's age, gender, region of residence within the U.S., race, education level, employment status, household income and whether the respondent voted for President Joseph Biden in the 2020 U.S. Presidential election. The behavioral controls include the types of news sources consumed, whether the respondent had low trust in the news media, the number of online services used, whether the respondent had signed a petition in the past 12 months, whether the respondent reported using one or more misinformation news outlets from a list of 26 popular news outlets in the past 12 months, the respondent's top choice gift card and whether the respondent frequently uses their top choice gift card company.

While we conduct our analyses for the subsample of participants who pass an attention check in our survey,

as a robustness check we present results for the full sample of respondents, including inattentive participants, in Supplementary Tables A9 and A10, which are qualitatively similar and remain statistically significant when compared to the results of the attentive sample.

1.2.2 Analysis of consumers' text responses

In our consumer survey, we ask our survey participants to briefly state the reason behind their choice of gift card and choice petition using an open-ended text field. we analyzed participants' text responses in order to understand their responses to each of these two behavioral outcomes. To do so, we first removed the names of companies from the text responses and then used the top2vec algorithm [99], which automatically outputs the number of clusters and assigns. Top2vec uses word embeddings that account for the context of a word in a document, which is an advantage this method has over bag-of-word approaches like Latent Dirichlet Allocation.

For our exit outcome, we observe six topics emerge from the algorithm. We manually inspect the responses and find responses in these clusters correspond to responses that mainly mention misinformation-related concerns, how much they like a given company's products, how much they love products from a given company, how much they use a given company, how frequently they use a given company and specific features of a company's products. We further cluster together responses that mention how much they like a company and how much they love a given company's products together into a single "product preference" cluster. Similarly, we merge together responses in the use and frequency of use clusters into a single "product usage" cluster. We end up with four main clusters as shown in Figure E1. Table A1 shows sample text responses belonging to each cluster. These clusters are as follows:

1. Misinformation: a binary variable that takes a value of 1 if a participant indicated companies ads appearing on misinformation websites as being a factor contributing to their final gift card choice and zero otherwise.
2. Product usage: a binary variable that takes value 1 when a participant mentions their use or frequency of use of the product and zero otherwise.
3. Product preference: a binary variable that takes value 1 if a respondent mentions how they or their family like, enjoy or love the product they chose and zero otherwise.
4. Product features: a binary variable that takes a value of 1 if a participants refers to specific features such as how convenient, healthy or close to home the product they chose is and zero otherwise.

For our voice outcome, we take the same approach as above. This process results in five key clusters, which are shown in Figure E1 with select sample text responses in Table A2. These clusters are as follows:

1. Company responsibility: a binary variable that takes a value of 1 if a participant's response indicated that companies are responsible for their ads appearing on misinformation websites and zero otherwise.
2. Platform responsibility: a binary variable that takes value of 1 if a participant's response indicated that digital ad platforms are responsible for companies' ads appearing on misinformation websites and zero

otherwise.

3. Misinformation concerns: a binary variable that takes value 1 if a participant's response mentions being concerned about misinformation and zero otherwise.

4. Best option: a binary variable that takes a value of 1 if a participant's explanation for their choice mentions the option they chose as being the best available option in their opinion and zero otherwise.

5. No interest: a binary variable that takes a value of 1 if a participant's response indicates that they would not like to sign an online petition and zero otherwise.

Table A1: Sample text responses from participants explaining their choice of gift card.

Sample	Text classification	Text response
1.	Misinformation	I will use a food delivery service more than using a driver service. I changed to grub hub because door dash allows their ads on websites with incorrect information.
2.	Misinformation	I first chose Uber as my choice because it is the only one that I use from the choices. However, I would happily switch to Lyft if their practices are more ethical.
3.	Misinformation	Subway was not a company that advertised on misinformation websites.
4.	Misinformation	I feel guilty about taking the burger king card if it is being used to further false information.
5.	Misinformation	I don't want to support the spread of misinformation.
6.	Misinformation	It was not listed among the sites that were linked to a misinformation site.
7.	Misinformation	I equally like door dash and grub hub but don't want to support a business business associated with misinformation.
8.	Product usage	I can use this to go to work.
9.	Product usage	Doordash is the only company out of these choices that I use on a regular basis.
10.	Product usage	I chose this card because over the past two years I have bought more subs then other food places.
11.	Product usage	Because i would most likely use this gift card on my next visit to Burger King and it is less likely that i would use the others.
12.	Product usage	I chose Burger King because it's the only restaurant and service I actually use from the above list.
13.	Product usage	I frequent this restaurant quite a bit, so it would be a good fit for me.
14.	Product usage	I chose the above gift card because it's the one that I'd get the most utility from.
15.	Product preference	This is one of my favorite fast food restaurants.
16.	Product preference	I love Burger King. There plenty of items on menu that are worth getting excited about. Yummy food.
17.	Product preference	I eat at Subway and I like the food.
18.	Product preference	The have a selection that I like with fast delivery.
19.	Product preference	I would like Doordash because it is my go to food app. I love that I get to choose from a variety of food restaurants and even for beverages. My children love it as well and that gift card is going to go to them.
20.	Product preference	This gift card is the one that will be most beneficial for my family.
21.	Product preference	Subway is mine and my children's favorite local restaurant. We love to "eat fresh" and at subway everything is always fresh and delicious!
22.	Product features	subway is good to eat because of the calories that are in the food.
23.	Product features	I personally use door dash quite a bit and it fits into the convenience of my life.
24.	Product features	Health choice and trying to be healthy.
25.	Product features	Subway has convinient locations and great food at good prices.
26.	Product features	I chose this one because it is a lot closer and there is a person at burger king i am trying to become friends with.
27.	Product features	I am in a rural area now where food delivery is non exsistent so I would like it only to take my family out.
28.	Product features	I chose this gift card because there is a Subway close enough that i can walk to. I dont have a vehicle to drive to burger king and I dont believe lyft and uber are offered here.

Table A2: Sample text responses from participants explaining their choice to sign an online petition.

Sample	Text classification	Text response
1.	Company responsibility	Companies like Subway absolutely should do this. The war on disinformation requires private and government action.
2.	Company responsibility	I think they should block their ads because of these misinformation sites causing their reputation harm.
3.	Company responsibility	Because it gives a bad reflection on the company and their brand if their ads are on websites that share misinformation.
4.	Company responsibility	All companies should be mindful of how they gain revenue and operate in society. Being ethical should always be at the forefront of their mission.
5.	Company responsibility	It can taint a company's image to be seen on misinformation websites.
6.	Platform responsibility	Because companies like subway depend on digital ad platforms to place their ads the responsibility lies with the ad platforms.
7.	Platform responsibility	Digital ad platforms should accept responsibility for placing ads on inappropriate and misleading sites.
8.	Platform responsibility	I feel like if we stop the use of ad platforms on misinformation sites in the first place then it would help out more in the long run.
9.	Platform responsibility	Digital ad platforms seem to make it easier to allow ads on misinformation websites.
10.	Platform responsibility	I feel that the onus is on digital ad platforms.
11.	Misinformation concerns	Supporting misinformation websites is horrible.
12.	Misinformation concerns	I do not want any misinformation sites to show ads.
13.	Misinformation concerns	Ads shouldn't help pay for misinformation.
14.	Misinformation concerns	I've always gotten misleading infotmation on multipule occasions and needs to stop.
15.	Misinformation concerns	No one should be supporting misinformation.
16.	Best option	It eliminates more of the problem than the others.
17.	Best option	sounded like the most plausible choice.
18.	Best option	It is the best way to cancel out their problem
19.	Best option	It is the right thing to do.
20.	Best option	This statement seems to address the problem on a more widespread basis.
21.	No interest	I have not seen any of these ads we are taking the survey about.
22.	No interest	Freedom of speech. Up to consumers to educate themselves via various platforms.
23.	No interest	Who decides what is misinformation. Today these claims may be true, but if legistaltion is enacted and it becomes what corporations or government disagree with, this subverts the first amendment.
24.	No interest	I am not interested in governing what people or companies advertise or report as news. They are within their right to do so. This is America and in America people have the right to be wrong. If they don't want to do the research to find if the information they are getting is false than that's also people's right to be lazy. It's unfortunate but true.
25.	No interest	I don't want to sigh the petition because its not for me to tell a company how or who to run their company ads whether i agree with it or not.

1.2.3 Decision-maker study outcomes

In Supplementary Table A12, we report summary statistics for our decision-maker sample and demonstrate that our treatment groups are balanced across observable characteristics.

In analyzing the effects of our information intervention, we run OLS regressions controlling for decision-makers' characteristics and beliefs.These controls include whether a decision-maker works in a full-time role, whether they work in a marketing role, the duration of their role at their company, the number of employees at their company, the industry of their company, and whether the company is headquartered in the U.S. Additionally, we control for the respondents' beliefs about companies and platforms advertising on misinformation, their knowledge of their company's use of digital ads, whether the respondent demands information about consumer backlash and whether they request an ad check.

As a robustness check, we also present results for all respondents, including inattentive respondents, in Supplementary Tables A14 and A15, which are statistically significant and qualitatively similar to the results reported in the paper in Tables 3 and 4, respectively.

2 Supplementary Tables and Figures

2.1 Descriptive results

This section contains further details on the data used for descriptive analysis. Table A3 shows summary statistics for the sample of domains obtained from NewsGuard.

Table A4 shows the number of unique companies by industry whose ads appear on the misinformation websites websites in our sample from 2019 to 2021.

In the paper, we further examine the top 100 most active advertisers during 2019-2021. A further segmentation of these advertisers reveals the following three groups of companies: 1) companies that used digital ad platforms each week throughout this period (20%), 2) companies that did not use digital ad platforms at all (24%), and 3) companies that both used and did not use digital ad platforms at least once during this period (56%). The companies in all three of these groups span a broad range of industries. However, those in the first group all appear on misinformation websites during this period (94% of the time) while none in the second group do so (barring one company, which appears on only one local misinformation website about 15% of the time it advertises) and those in the third group appear on misinformation websites about 47% of the time on average. To take a closer look at companies in the third group above that appear on misinformation sites without using platforms, we filter the companies in this group to those that both use and do not use digital ad platforms in at least a quarter of the three-year period to ensure a meaningful comparison. This results in a small subsample that constitutes 7% of the overall sample of the top 100 most active advertisers, of which all except one appear on misinformation websites at least once when not using platforms. All of these few companies belong to Media & Entertainment or Publishing industries (with one company belonging to the Online Services category). Importantly, even within this small group of companies, the majority of appearances on misinformation occur in weeks companies are using digital ad platforms.

Table A3: Summary statistics for NewsGuard data

	US		Global	
	Misinformation	Non-misinformation	Misinformation	Non-misinformation
Average score	17.3	73.7	17.9	76.6
% of trustworthy websites	6.1	70.1	6.0	76.4
% of websites with paywall	2.7	25.0	3.2	24.0
% of owned by individuals	25.3	4.0	27.1	3.8
% of owned by governments	1.1	0.4	2.1	1.1
% of owned by private companies	19.1	60.0	21.6	60.1
% of owned by public companies	1.4	24.7	1.3	25.2
% of owned by non-profits	4.8	7.2	6.3	6.4
% of neutral websites	19.5	68.8	23.3	73.3
% of right-wing websites	76.9	24.5	72.7	19.6
% of left-wing websites	3.5	6.8	4.1	7.1
Observations	1449	4838	1745	6499

Notes: These summary statistics are based on data provided by NewsGuard. NewsGuard assigns an aggregated score from 0 to 100 to each website based on a weighted average of how well it performs on its nine journalistic criteria, and considers websites that receive a rating below 60 to be untrustworthy websites. *Average score* shows the mean NewsGuard score and *% of untrustworthy websites* refers to NewsGuards' classification.

Table A4: Number of companies whose ads appear on misinformation websites

Industry	N
Holding Companies	6767
Online Services	5347
Media	4749
Technology	4157
Govt., Politics or Religion	3851
Business Solutions	3848
Household	3644
Travel	3484
Apparel	3373
Retail	3368
Insurance	3307
Telecommunications	3189
Digital Publishing	3111
Print Publishing	3103
Finance	3018
Health	2980
Babies & Kids	2344
Automotive	1766
Food or Beverages	1688
Industrial	1180
Education	1032
Dining	1028
Gas & Electric	457
Cosmetics	340
Arms	28

Notes: This table shows the number of unique companies whose ads appear on misinformation websites between 2019 and 2021 for each of the 25 industries in the Moat Pro dataset. Some companies belong to more than one industry.

2.2 Consumer study results

2.2.1 Summary statistics and analyses

Table A5: Summary statistics and balance across treatment arms for the consumer survey.

	All	Information treatments					
	(1)	(2)	(3)	(4)	(5)	(6)	(7)
		Control	T1	T2	T3	T4	p-value
Duration (in seconds)	1185	1005	1095	1032	1669	1122	0.14
Gender (Female)	0.52	0.53	0.50	0.53	0.55	0.49	0.17
Gender (Male)	0.47	0.46	0.49	0.46	0.45	0.51	0.13
Race (White)	0.78	0.82	0.78	0.77	0.77	0.77	0.07
Age (Below 45)	0.45	0.44	0.46	0.44	0.46	0.45	0.90
Residence (North East)	0.18	0.18	0.19	0.20	0.18	0.17	0.52
Residence (Midwest)	0.21	0.20	0.21	0.20	0.21	0.22	0.85
Residence (South)	0.40	0.40	0.41	0.39	0.39	0.40	0.90
Residence (West)	0.21	0.22	0.19	0.20	0.22	0.21	0.58
Household income ($<$ 50K)	0.46	0.48	0.48	0.46	0.44	0.46	0.49
Education (No degree)	0.47	0.48	0.48	0.47	0.45	0.46	0.85
Education (At least college)	0.41	0.40	0.40	0.41	0.44	0.40	0.43
Employment (Working)	0.52	0.50	0.51	0.52	0.52	0.53	0.69
Employment (Not working)	0.47	0.49	0.48	0.46	0.47	0.46	0.76
Partisanship (Democrat)	0.44	0.42	0.43	0.47	0.47	0.42	0.12
Partisanship (Republican)	0.32	0.33	0.33	0.31	0.30	0.33	0.51
Vote (Trump)	0.32	0.33	0.33	0.31	0.30	0.35	0.14
Vote (Biden)	0.47	0.47	0.47	0.49	0.48	0.42	0.10
Vote (Other)	0.03	0.02	0.04	0.03	0.03	0.05	0.13
Vote (None)	0.18	0.17	0.17	0.17	0.19	0.18	0.75
Frequent user	0.57	0.53	0.57	0.58	0.56	0.61	0.03
Infrequent user	0.18	0.20	0.19	0.17	0.18	0.18	0.53
Prior petitions signed	0.35	0.35	0.33	0.37	0.35	0.36	0.64
Consumes misinformation	0.30	0.28	0.30	0.29	0.29	0.32	0.48
Media trust (Low)	0.34	0.36	0.33	0.33	0.33	0.34	0.78
Media trust (High)	0.25	0.25	0.27	0.25	0.24	0.24	0.50
Observations	4039	806	808	802	809	814	

Notes: The p-values reported in Column (7) are derived from two-sided t-tests. No adjustments were made for multiple comparisons.

Table A6: P-values for Pairwise Treatment Comparisons

	Switch in preference		Switch to lower preference		Switch in category		Switch to lower misinformation	
	(1)	(2)	(3)	(4)	(5)	(6)	(7)	(8)
P-value (T1 vs T2)	< 0.001	< 0.001	< 0.001	< 0.001	0.004	0.002	0.176	0.247
P-value (T1 vs T3)	0.083	0.052	0.197	0.150	0.440	0.338	0.259	0.091
P-value (T1 vs T4)	0.003	0.002	0.074	0.067	0.052	0.031	0.084	0.289
P-value (T2 vs T3)	< 0.001	< 0.001	< 0.001	< 0.001	0.033	0.036	0.662	0.894
P-value (T2 vs T4)	< 0.001	< 0.001	< 0.001	< 0.001	0.332	0.340	0.009	0.077
P-value (T3 vs T4)	0.217	0.270	0.623	0.706	0.241	0.240	0.008	0.008
Controls	No	Yes	No	Yes	No	Yes	No	Yes
Observations	4039	4039	4039	4039	4039	4039	430	430

$^{***}p < 0.01$, $^{**}p < 0.05$, $^{*}p < 0.1$

Notes: This table shows the remaining pairwise p-values from the OLS regression results reported in Table 1. These p-values were derived from two-sided t-tests. No adjustments were made for multiple comparisons.

Table A7: Average Treatment Effects on Voice

	Company		Platform	
	(1)	(2)	(3)	(4)
P-value (T1 vs T2)	0.052	0.056	< 0.001	< 0.001
P-value (T1 vs T3)	0.130	0.225	0.550	0.575
P-value (T1 vs T4)	0.518	0.408	0.495	0.531
P-value (T2 vs T3)	0.665	0.481	0.002	0.002
P-value (T2 vs T4)	0.010	0.006	< 0.001	< 0.001
P-value (T3 vs T4)	0.031	0.040	0.201	0.234
Controls	No	Yes	No	Yes
Control group mean	0.15	0.15	0.14	0.14
Observations	4039	4039	4039	4039

$^{***}p < 0.01$, $^{**}p < 0.05$, $^{*}p < 0.1$

Notes: This table shows the remaining pairwise p-values from the OLS regression results reported in Table 2. These p-values were derived from two-sided t-tests. No adjustments were made for multiple comparisons.

Figure A3: Participants' stated and revealed responses in terms of (a) exit and (b) voice.

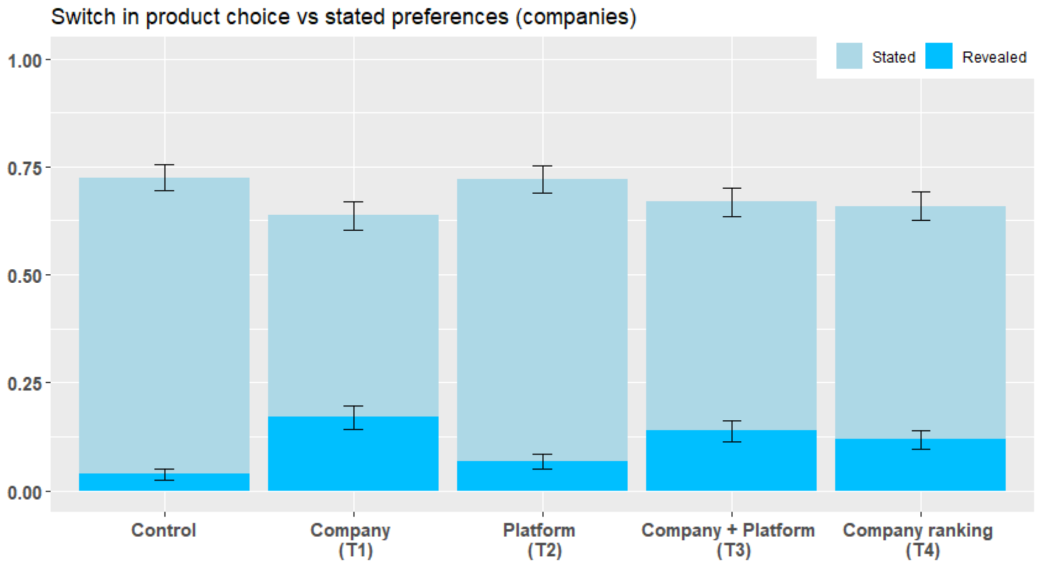

(a) This figure shows participants' revealed preferences against their stated preferences regarding the role of advertising companies in financing misinformation ($n = 4039$). Data are presented as mean values with the vertical bars representing 95% confidence intervals derived from standard errors. Revealed preferences are measured by the proportion of participants in each group who switch their gift card choice (i.e. "exit") after receiving the information treatment. Stated responses show the proportion of participants' who agree or strongly agree with the statement "Companies have an important role to play in reducing the spread of misinformation through their advertising practices". This comparison should be viewed as suggestive since the aforementioned stated preference question does not exactly map into the revealed preference question about gift card choice.

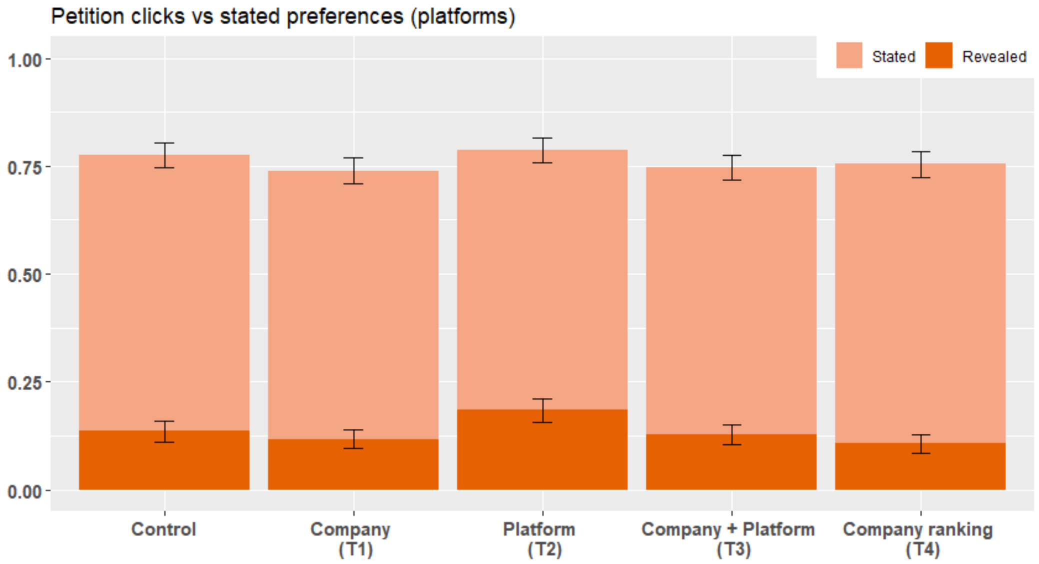

(b) This figure shows participants' revealed preferences against their stated preferences regarding the role of digital ad platforms in financing misinformation ($n = 4039$). Data are presented as mean values with the vertical bars representing 95% confidence intervals derived from standard errors. Revealed preferences are measured by the proportion of participants in each group who click on a link to sign a petition suggesting that digital ad platforms should block ads on misinformation websites. Stated responses show the proportion of participants' who agree or strongly agree with the statement "Digital platforms should give companies the option to avoid advertising on misinformation websites." This comparison should be viewed as suggestive since the aforementioned stated preference question does not exactly map into the revealed preference question about signing an online petition.

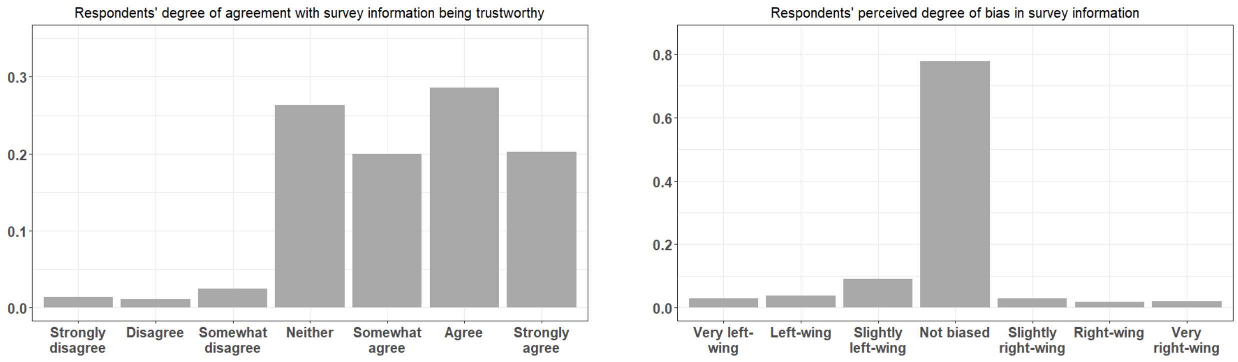

(a) Distribution of participants' responses to the question "The information provided in this survey is trustworthy."

(b) Distribution of participants' responses to the question "Do you think that this survey was biased?"

Figure A4: Participants' perception of survey information.

2.2.2 Robustness checks

Table A8: Heterogeneity in switching given difference in weights assigned to initial and final choice gift card

	Switch in preference		
	(1)	(2)	(3)
Treatment	0.12^{***}	0.11^{***}	0.11^{***}
	(0.01)	(0.01)	(0.01)
	< 0.001	< 0.001	< 0.001
Treatment × Weight difference	-0.001^{***}		
	(0.00)		
	< 0.001		
Treatment × Weight difference (> Mean)		-0.06^{***}	
		(0.02)	
		< 0.001	
Treatment × Weight difference (> Median)			-0.05^{**}
			(0.02)
			0.007
Weight difference	-0.001^{***}		
	(0.00)		
	0.002		
Weight difference (> Mean)		-0.03^{***}	
		(0.01)	
		0.009	
Weight difference (> Median)			-0.03^{***}
			(0.01)
			0.005
Controls	Yes	Yes	Yes
Observations	4039	4039	4039

$^{***}p < 0.01$, $^{**}p < 0.05$, $^{*}p < 0.1$

Notes: This table shows OLS regressions where the dependent variable, *switch in preference*, is the same primary exit outcome shown in Table 1 in the paper ($n = 4039$). *Treatment* is a pooled binary variable, which takes value 1 when a respondent is randomly assigned to receive any of our information treatments (i.e., T1, T3 and T4) that mention their top choice gift card company advertising on misinformation websites. The interaction term *Weight difference* is a continuous variable for the difference between the participant's highest weighted gift card option and the second highest weighted gift card. The terms *Weight difference > Mean* and *Weight difference > Median* are binary variables that take value 1 when this difference exceeds the mean weight difference (31.7) and the median weight difference (20), respectively, and zero otherwise. No adjustments were made for multiple comparisons. Robust standard errors in parentheses. P-values derived from two-sided t-tests reported below standard errors.

Table A9: Robustness Check Including Inattentive Respondents: Average Treatment Effects on Exit

	Switch in preference		Switch to lower preference		Switch in category		Switch to lower misinformation	
	(1)	(2)	(3)	(4)	(5)	(6)	(7)	(8)
Company (T1)	0.07^{***}	0.06^{***}	0.04^{***}	0.04^{***}	0.02^{***}	0.02^{***}	0.40^{*}	0.27
	(0.01)	(0.01)	(0.01)	(0.01)	(0.01)	(0.01)	(0.23)	(0.23)
	< 0.001	< 0.001	< 0.001	< 0.001	0.002	0.004	0.085	0.231
Platform (T2)	0.01	0.01	0.01	0.00	0.01	0.00	-0.26	-0.19
	(0.01)	(0.01)	(0.01)	(0.01)	(0.01)	(0.01)	(0.26)	(0.26)
	0.273	0.518	0.372	0.474	0.261	0.501	0.319	0.476
Company and Platform (T3)	0.07^{***}	0.07^{***}	0.05^{***}	0.05^{***}	0.03^{***}	0.03^{***}	0.27	0.19
	(0.01)	(0.01)	(0.01)	(0.01)	(0.01)	(0.01)	(0.23)	(0.23)
	< 0.001	< 0.001	< 0.001	< 0.001	< 0.001	< 0.001	0.242	0.409
Company Ranking (T4)	0.05^{***}	0.04^{***}	0.03^{***}	0.03^{***}	0.02^{***}	0.02^{**}	0.74^{***}	0.56^{**}
	(0.01)	(0.01)	(0.01)	(0.01)	(0.01)	(0.01)	(0.24)	(0.23)
	< 0.001	< 0.001	< 0.001	< 0.001	0.008	0.022	0.002	0.015
Controls	No	Yes	No	Yes	No	Yes	No	Yes
Control group mean	0.08	0.08	0.04	0.04	0.05	0.05	1.20	1.20
Observations	9648	9648	9648	9648	9648	9648	1153	1153

$^{***}p < 0.01$, $^{**}p < 0.05$, $^{*}p < 0.1$

Notes: This table reproduces OLS regressions for all specifications shown in Table 1 for the full sample of participants, including inattentive respondents ($n = 9648$). In Columns (2), (4), (6), and (8), we use the same control variables detailed in Supplementary Information, "Analysis: Consumer study outcomes" except for race, political orientation, education level, employment status and household income since these variables were collected after inattentive participants were screened out of the study. No adjustments were made for multiple comparisons. Robust standard errors in parentheses. P-values derived from two-sided t-tests reported below standard errors.

Table A10: Robustness Check Including Inattentive Respondents: Average Treatment Effects on Voice (Intention to Sign a Petition)

	Original Sample: Attentive Respondents				All Respondents (Including Inattentive)			
	Company		Platform		Company		Platform	
	(1)	(2)	(3)	(4)	(5)	(6)	(7)	(8)
Company (T1)	0.04^{*}	0.03^{*}	-0.02	-0.02	0.02	0.02	-0.01	-0.01
	(0.02)	(0.02)	(0.02)	(0.02)	(0.01)	(0.01)	(0.01)	(0.01)
	0.066	0.095	0.441	0.416	0.101	0.117	0.281	0.239
Platform (T2)	0.01	0.01	0.05^{**}	0.05^{**}	-0.00	-0.00	0.03^{**}	0.03^{**}
	(0.02)	(0.02)	(0.02)	(0.02)	(0.01)	(0.01)	(0.01)	(0.01)
	0.638	0.800	0.014	0.020	0.958	0.806	0.021	0.038
Company and Platform (T3)	0.01	0.01	0.03	0.03	0.01	0.01	0.01	0.01
	(0.02)	(0.02)	(0.02)	(0.02)	(0.01)	(0.01)	(0.01)	(0.01)
	0.580	0.611	0.201	0.215	0.377	0.441	0.262	0.243
Company Ranking (T4)	0.05^{**}	0.04^{*}	-0.03	-0.03	0.03^{**}	0.03^{**}	-0.01	-0.01
	(0.02)	(0.02)	(0.02)	(0.02)	(0.01)	(0.01)	(0.01)	(0.01)
	0.028	0.068	0.108	0.075	0.025	0.039	0.624	0.426
Controls	No	Yes	No	Yes	No	Yes	No	Yes
Control mean	0.22	0.22	0.21	0.21	0.20	0.20	0.18	0.18
Observations	4039	4039	4039	4039	9648	9648	9648	9648

$^{***}p < 0.01$, $^{**}p < 0.05$, $^{*}p < 0.1$

Notes: This table shows OLS regressions where the dependent variable is intention to sign a given petition, a binary variable that takes the value 1 when a participant indicates wanting to sign a given petition and zero otherwise. Columns (1)-(4) show results for attentive participants ($n = 4039$) and Columns (5)-(8) show results for all participants, including inattentive ones ($n = 9648$). We use the intention to sign outcome to compare attentive and inattentive participants since the inattentive participants were screened out of the study before data for the other petition outcomes was calculated. Columns (1)-(2) and (5)-(6) refer to company-specific petitions suggesting that companies like the respondent's top choice gift card company need to block their ads from appearing on misinformation websites. Columns (3)-(4) and (7)-(8) refer to platform-specific petitions suggesting that digital ad platforms used by companies need to block ads from appearing on misinformation websites. In Columns (2), (4), (6), and (8), we use the same control variables detailed in Supplementary Information, "Analysis: Consumer study outcomes" except for race, political orientation, education level, employment status and household income since these variables were collected after inattentive participants were screened out of the study. No adjustments were made for multiple comparisons. We apply robust standard errors in parentheses and report p-values derived from two-sided t-tests below standard errors.

Table A11: Treatment prediction confusion matrices for the consumer experiment

	Predicted Control	Predicted Treated
**Panel A: Control vs. T1**		
True Control	92	107
True Treated	90	115
		Overall accuracy: 51.2%
**Panel B: Control vs. T2**		
True Control	106	92
True Treated	104	100
		Overall accuracy: 51.2%
**Panel C: Control vs. T3**		
True Control	106	91
True Treated	101	106
		Overall accuracy: 52.5%
**Panel D: Control vs. T4**		
True Control	87	123
True Treated	82	113
		Overall accuracy: 49.4%

Notes: This table presents the confusion matrices for the study purpose responses by participants in our consumer experiment. Each cell counts the number of participants assigned to the randomized group in the row and classified by the Support Vector Machine to be in the randomized group in the column.

2.3 Decision-maker study results

2.3.1 Summary statistics and analyses

Table A12: Summary Statistics for Decision-maker Study

		Full sample<br>(1)	Treated<br>(2)	Control<br>(3)	P-value<br>(4)
	Full-time employee	0.72	0.71	0.74	0.473
	Marketing role	0.10	0.08	0.11	0.252
Characteristics	Gender (Female)	0.21	0.21	0.20	0.808
	Duration in role (>5 years)	0.58	0.60	0.57	0.512
	Number of employees (>100)	0.59	0.55	0.63	0.080
	Headquartered in the U.S.	0.43	0.45	0.42	0.483
	Estimated consumer backlash	0.41	0.40	0.42	0.708
	Company beliefs	64.3	65.8	62.9	0.308
Beliefs	Prior platform beliefs	53.0	53.5	52.5	0.773
	Own company belief (yes)	0.20	0.19	0.21	0.634
	Own company belief uncertainty	0.21	0.23	0.19	0.288
	Misinformation control	0.88	0.87	0.88	0.641
	Company responsibility	0.76	0.80	0.73	0.062
Stated preferences	Platform responsibility	0.86	0.88	0.84	0.239
	Stated ad check demand	0.76	0.88	0.84	0.133
	Stated solution demand	0.71	0.72	0.69	0.598
Observations		442	216	226	

Notes: This table shows descriptive statistics for our sample of decision-makers ($n = 442$). *Estimated consumer backlash* is a binary variable that takes the value 1 if a participant agrees or strongly agrees that "Consumers react against companies whose ads appear on misinformation websites" and zero otherwise. *Company beliefs* is the estimated proportion of companies whose ads appear on misinformation websites. *Prior platform beliefs* is the estimated proportion of companies that use digital ad platforms and whose ads on appear on misinformation websites. *Own company belief (yes)* is the proportion of respondents who report "yes" when asked whether they believe their own company's ads appeared on misinformation websites. *Own company belief uncertainty* is the proportion of respondents who report being uncertain (unsure or very unsure) about their response to the aforementioned company belief question. *Misinformation control* is a binary variable that takes the value 1 if a participant agrees or strongly agrees that "It is important to control the spread of misinformation in society" and zero otherwise. *Company responsibility* is a binary variable that takes the value 1 if a participant agrees or strongly agrees that "Companies have an important role to play in reducing the spread of misinformation through their advertising practices" and zero otherwise. *Platform responsibility* is a binary variable that takes the value 1 if a participant agrees or strongly agrees that "Digital platforms should give companies the option to avoid advertising on misinformation websites" and zero otherwise. *Stated ad check demand* is a binary variable that takes the value 1 if a participant agrees or strongly agrees to the statement "I would like to find out whether my company's ads are appearing on misinformation websites" and zero otherwise. *Stated solution demand* is a binary variable that takes the value 1 if a participant agrees or strongly agrees to the statement "I would recommend that my company adopt a product to avoid advertising on misinformation websites" and zero otherwise. The p-values reported in Column (4) are derived from two-sided t-test comparison of means between the treated (Column 2) and control (Column 3) groups.

Figure A5: Characteristics of the Decision-maker Sample

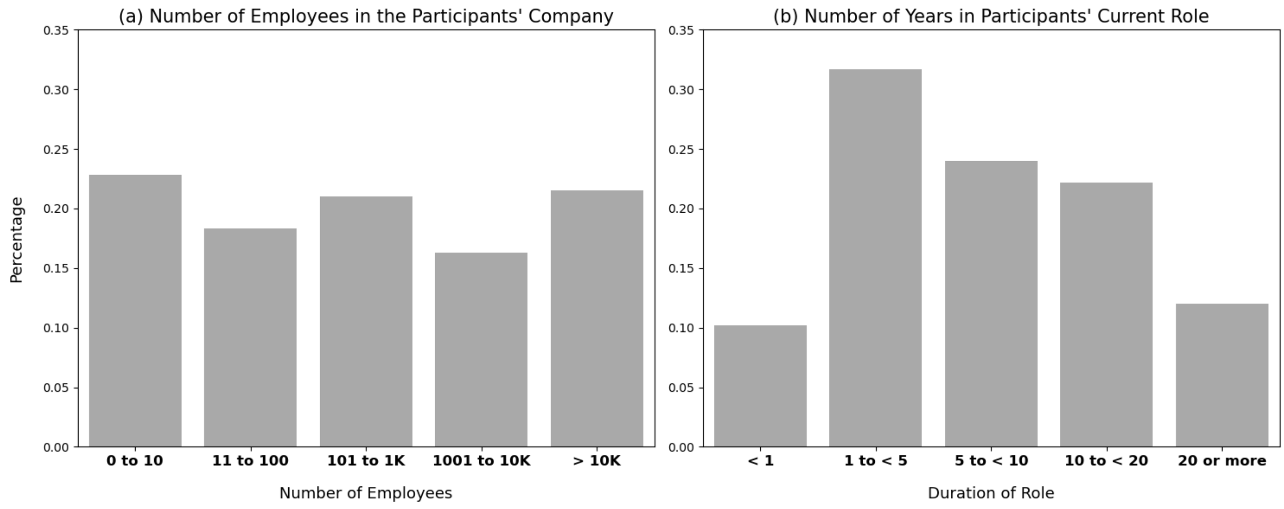

Figure A6: Distribution of Beliefs About Advertising Companies

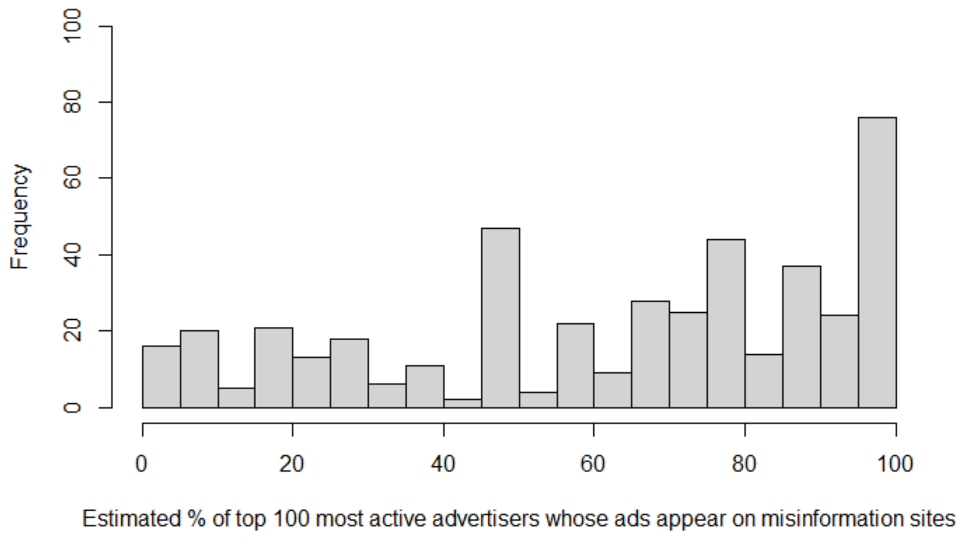

Figure A7: Distribution of Beliefs About Digital Ad Platforms

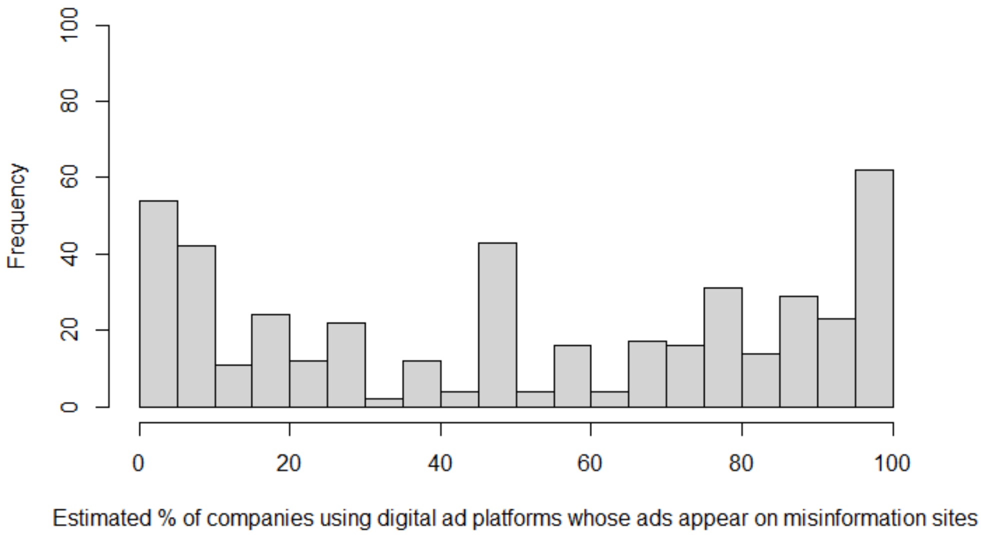

Table A13: Treatments Effects on Donation to the Global Disinformation Index (GDI)

	Full sample	Prior belief: No		
		All	Certain	Uncertain
	(1)	(2)	(3)	(4)
Treatment	−0.01	−0.01	−0.03	0.01
	(0.05)	(0.05)	(0.06)	(0.17)
	0.757	0.911	0.655	0.935
Controls	Yes	Yes	Yes	Yes
Control group mean	0.60	0.60	0.59	0.67
Observations	442	354	286	68

$^{***}p < 0.01$, $^{**}p < 0.05$, $^{*}p < 0.1$

Notes: This table shows OLS regression results where the dependent variable is donation to the Global Disinformation Index or GDI, a binary variable that takes a value of one when a participant chooses to donate to GDI and zero when a participant chooses to donate to DataKind, the alternative charity option provided. Column (1) shows results for the full sample of participants ($n = 442$) and columns (2)-(4) show results for the sub-sample of participants who reported "No" to the question "Do you think your company or organization had its ads appear on misinformation websites during the past three years (2019-2021)?" ($n = 354$). In column (2), we report results for all participants who reported "No" to the aforementioned question. Column (3) shows results for participants who report being certain about their response to the aforementioned question, i.e. choosing "Somewhat sure", "Sure" or "Very sure" ($n = 286$). Column (4) shows results for participants who report being uncertain about their response to the aforementioned question, i.e.choosing "Unsure" or "Very unsure" ($n = 68$). We control for decision-makers' characteristics and prior beliefs in all specifications. No adjustments were made for multiple comparisons. Robust standard errors in parentheses. P-values derived from two-sided t-tests reported below standard errors.

2.3.2 Robustness checks

Table A14: Robustness Check Including Inattentive Respondents: Average Treatment Effects of Information Intervention

	Posterior belief			Platform solution demand		
	All	Yes	No	All	Yes	No
	(1)	(2)	(3)	(4)	(5)	(6)
Treatment	54.60^{**}	29.58	49.96^{**}	-0.02	-0.09	-0.02
	(22.20)	(69.55)	(24.83)	(0.04)	(0.12)	(0.05)
	0.014	0.672	0.036	0.714	0.440	0.641
Controls	Yes	Yes	Yes	Yes	Yes	Yes
Control group mean	121.30	150.83	113.44	0.36	0.30	0.37
Observations	508	107	401	508	107	401

$^{***}p < 0.01$, $^{**}p < 0.05$, $^{*}p < 0.1$

Notes: This table replicates the OLS regressions reported in Table 3 for all participants, including those whose responses to our survey questions suggested that they were inattentive during our survey ($n = 508$). No adjustments were made for multiple comparisons. Robust standard errors in parentheses. P-values derived from two-sided t-tests reported below standard errors.

Table A15: Robustness Check Including Inattentive Respondents: Treatments Effects Based On Prior Beliefs

	Posterior belief		Platform solution demand	
	Certain	Uncertain	Certain	Uncertain
	(1)	(2)	(3)	(4)
Treatment	29.73	158.26^{**}	-0.05	0.29^{**}
	(28.93)	(61.58)	(0.06)	(0.13)
	0.305	0.014	0.359	0.038
Controls	Yes	Yes	Yes	Yes
Control group mean	117.76	91.18	0.35	0.48
Observations	326	75	326	75

$^{***}p < 0.01$, $^{**}p < 0.05$, $^{*}p < 0.1$

Notes: Notes: This table replicates the OLS regressions reported in Table 4 for all participants, including those whose responses to our survey questions suggested that they were inattentive during our survey ($n = 401$). No adjustments were made for multiple comparisons. Robust standard errors in parentheses. P-values derived from two-sided t-tests reported below standard errors.

Table A16: Treatment prediction confusion matrices for the decision-maker experiment

	Predicted Control	Predicted Treated
True Control	22	23
True Treated	23	21
		Overall accuracy: 48.3%

Notes: This table presents the confusion matrix for the study purpose responses by participants in our decision-maker experiment. Each cell counts the number of participants assigned to the randomized group in the row and classified by the Support Vector Machine to be in the randomized group in the column.

Table A17: Summary Statistics for Verified and Self-reported Decision-makers

		Verified (1)	Self-reported (2)	P-value (3)
Characteristics	Full-time employee	0.74	0.75	0.848
	Marketing role	0.07	0.11	0.038
	Gender (Female)	0.21	0.21	0.976
	Duration in role (> 5 years)	0.62	0.60	0.575
	Number of employees (> 100)	0.60	0.60	0.987
	Headquartered in the U.S.	0.42	0.43	0.738
Beliefs	Estimated consumer backlash	0.42	0.43	0.795
	Company beliefs	64.0	64.3	0.902
	Prior platform beliefs	53.2	52.4	0.783
	Own company belief (yes)	0.20	0.20	0.981
	Own company belief uncertainty	0.23	0.21	0.471
Stated preferences	Misinformation control	0.88	0.89	0.693
	Company responsibility	0.77	0.77	0.925
	Platform responsibility	0.87	0.87	0.749
	Stated ad check demand	0.78	0.79	0.735
	Stated solution demand	0.73	0.74	0.616
Observations		297	341	

Notes: This table shows descriptive statistics for the decision-makers (i.e. individuals holding executive or managerial roles) in our second experiment. Column (1) represents respondents whose job titles we verified from external sources, e.g., LinkedIn, Crunchbase, etc. ($n = 297$). Column (2) represents respondents who self-reported their job titles ($n = 341$). Column (3) shows the p-value obtained via a two-sided t-test between two groups in (2) and (3). The variables shown here are the same as those in Table A12.

Table A18: Average Treatment Effects of Information Intervention for Sub-sample.

	Posterior belief			Platform solution demand		
	All	Yes	No	All	Yes	No
	(1)	(2)	(3)	(4)	(5)	(6)
Treatment	52.10^{***}	-14.69	61.93^{***}	-0.04	-0.15	-0.04
	(15.73)	(54.67)	(17.26)	(0.05)	(0.15)	(0.06)
	0.001	0.789	0.001	0.474	0.320	0.517
Controls	Yes	Yes	Yes	Yes	Yes	Yes
Observations	395	80	315	395	80	315

$^{***}p < 0.01$, $^{**}p < 0.05$, $^{*}p < 0.1$

Notes: This table shows OLS regression results corresponding to Table 3 in the paper for the sub-sample of participants in managerial and executive roles ($n = 395$). To construct this subsample, we drop non-executive and non-manager participants for the 315 participants where we have verified job titles. For the remainder participants with only self-reported titles available, we drop participants who self-reported a role other than an executive or managerial role. No adjustments were made for multiple comparisons. Robust standard errors in parentheses. P-values derived from two-sided t-tests reported below standard errors.

Table A19: Treatments Effects Based On Prior Beliefs for Sub-sample

	Posterior belief		Platform solution demand	
	Certain	Uncertain	Certain	Uncertain
	(1)	(2)	(3)	(4)
Treatment	54.53^{***}	132.14^{**}	-0.10	0.35^{**}
	(19.12)	(60.29)	(0.06)	(0.13)
	0.005	0.036	0.124	0.011
Controls	Yes	Yes	Yes	Yes
Observations	252	63	252	63

$^{***}p < 0.01$, $^{**}p < 0.05$, $^{*}p < 0.1$

Notes: This table shows OLS regression results corresponding to Table 4 in the paper for the sub-sample of participants in managerial and executive roles ($n = 315$). Similar to Table A18 above, we construct this sub-sample by dropping non-executive and non-manager participants for the 315 participants where we have verified job titles. For the remainder participants with only self-reported titles available, we drop participants who self-reported a role other than an executive or managerial role. No adjustments were made for multiple comparisons. Robust standard errors in parentheses. P-values derived from two-sided t-tests reported below standard errors.

References

99. Angelov, D. Top2Vec: Distributed Representations of Topics. Retrieved from: https://arxiv.org/abs/2008.09470v1 (Aug. 2020).
