# Supplementary material for: Companies inadvertently fund online misinformation despite consumer backlash
Source: Nature. 2024 Jun 5;630(8015):123–31. doi: 10.1038/s41586-024-07404-1 (PMC11153133; doi:10.1038/s41586-024-07404-1)
Supplement: Supplementary file 2 — Reporting Summary [file 41586_2024_7404_MOESM2_ESM.pdf]

Corresponding author(s): Wajeeha Ahmad

Last updated by author(s): Mar 22, 2023

Reporting Summary

Nature Portfolio wishes to improve the reproducibility of the work that we publish. This form provides structure for consistency and transparency in reporting. For further information on Nature Portfolio policies, see our Editorial Policies and the Editorial Policy Checklist.

Statistics

For all statistical analyses, confirm that the following items are present in the figure legend, table legend, main text, or Methods section.

n/a	Confirmed	
☐	☒	The exact sample size (*n*) for each experimental group/condition, given as a discrete number and unit of measurement
☐	☒	A statement on whether measurements were taken from distinct samples or whether the same sample was measured repeatedly
☐	☒	The statistical test(s) used AND whether they are one- or two-sided<br>*Only common tests should be described solely by name; describe more complex techniques in the Methods section.*
☐	☒	A description of all covariates tested
☐	☒	A description of any assumptions or corrections, such as tests of normality and adjustment for multiple comparisons
☐	☒	A full description of the statistical parameters including central tendency (e.g. means) or other basic estimates (e.g. regression coefficient) AND variation (e.g. standard deviation) or associated estimates of uncertainty (e.g. confidence intervals)
☐	☒	For null hypothesis testing, the test statistic (e.g. *F*, *t*, *r*) with confidence intervals, effect sizes, degrees of freedom and *P* value noted<br>*Give P values as exact values whenever suitable.*
☒	☐	For Bayesian analysis, information on the choice of priors and Markov chain Monte Carlo settings
☐	☒	For hierarchical and complex designs, identification of the appropriate level for tests and full reporting of outcomes
☐	☒	Estimates of effect sizes (e.g. Cohen's *d*, Pearson's *r*), indicating how they were calculated

Our web collection on statistics for biologists contains articles on many of the points above.

Software and code

Policy information about availability of computer code

Data collection	Data on advertising was obtained using Oracle's Moat Pro platform, which collects data by scraping thousands of websites each day. Data on companies and ad platforms was manually extracted from this platform from January 1, 2019 to December 31, 2021. Data collection for both survey experiments was done using the university provided Qualtrics survey software. Code supporting the findings of the paper is available at: https://github.com/wajeeha-ahmad/misinformation-advertising.
Data analysis	Data was cleaned and partially analyzed in Python using Jupyter notebooks. Analysis was completed using R version 4.0.3.

For manuscripts utilizing custom algorithms or software that are central to the research but not yet described in published literature, software must be made available to editors and reviewers. We strongly encourage code deposition in a community repository (e.g. GitHub). See the Nature Portfolio guidelines for submitting code & software for further information.

Data

Policy information about availability of data

All manuscripts must include a data availability statement. This statement should provide the following information, where applicable:

- Accession codes, unique identifiers, or web links for publicly available datasets
- A description of any restrictions on data availability
- For clinical datasets or third party data, please ensure that the statement adheres to our policy

Our study was preregistered at the American Economic Association's Registry under AEARCTR-0009973. The data we collected for our experimental studies is

available in anonymized form and can be accessed by clicking on this link: https://github.com/wajeeha-ahmad/misinformation-advertising. Data on job titles for the second survey experiment are not available to protect participant confidentiality. Data analyzing the descriptive analysis of advertising on misinformation websites can be made available after obtaining permission from the proprietary sources on misinformation domains (NewsGuard and the Global Disinformation Index) and advertising (Oracle).

Research involving human participants, their data, or biological material

Policy information about studies with human participants or human data. See also policy information about sex, gender (identity/presentation), and sexual orientation and race, ethnicity and racism.

Reporting on sex and gender	Participants in both surveys were asked to self-report their gender as part of the survey using a single question (Q. What is your gender? Response categories: Male, Female, Non-binary or third gender, Prefer not to say). Gender representation was considered for the consumer survey to ensure a nationally representative sample of the U.S. population based on gender among other dimensions. While our findings apply to all participants regardless of gender, we report pre-registered gender-specific results for our consumer experiment in the section titled "Heterogeneous treatment effects". Gender-based analysis was not performed for the decision-maker study given its small sample size. Our consumer sample consisted of 52% female participants and our decision-maker sample consisted of 21% female participants.
Reporting on race, ethnicity, or other socially relevant groupings	Participants in our consumer survey were asked to self-report their race or ethnicity using a single survey question (Q: which of the following best describes your ethnicity or race? Response categories: Asian/Asian American, Caucasian/White, Native American/Inuit/Aleut, Native Hawaiian/Pacific Islander, Other, Prefer not to say.) Race was considered in the survey design to ensure a nationally representative sample of the U.S. population in terms of race among other dimensions. We also controlled for the respondents' race category chosen in response to the above question while performing our analyses. No data on race was collected for the decision-maker study.
Population characteristics	See the "Behavioral and social sciences study design" responses below. Population characteristics are further detailed in the Methods section of our paper. Summary statistics for our participant populations are also reported in Supplementary Information Tables A5 and A11.
Recruitment	Participants in our consumer survey were recruited via CloudResearch. These participants were invited to "take a survey about the news, technology and businesses." This generic description does not make any specifc references to misinformation or its effects to avoid self-selection based on interest in or perceptions of misinformation. Participants may have self-selected into taking our survey based on their broad interest in news, technology and/or businesses; given the expected prevalence of such general interests among the consumers of the advertising companies we study, we do not expect such self-selection to substantially bias our results.<br><br>Participants in our decision-maker survey were recruited via emails sent by our partner organizations. We used neutral language in our study recruitment emails to attract a broad audience of participants to our survey regardless of their initial beliefs and concerns about misinformation, stating our goal as "conducting vital research on the role of digital technologies in impacting your organization" without mentioning misinformation. While our sample is limited to those decision-makers who participated in executive education programs at our two partner programs, we find the industries these decision-makers were from to be representative of the industries we observed in our descriptive analysis of advertisers appearing on various misinformation websites. Further details about the representativeness and validity of our sample are provided in Methods, "Decision-maker experiment design: Tackling experimental validity concerns".
Ethics oversight	The study was reviewed by the Stanford University Institutional Review Board (Protocol No. IRB-63897) and the Carnegie Mellon University Institutional Review Board (Protocol No. IRB00000603).

Note that full information on the approval of the study protocol must also be provided in the manuscript.

Field-specific reporting

Please select the one below that is the best fit for your research. If you are not sure, read the appropriate sections before making your selection.

☐ Life sciences ☒ Behavioural & social sciences ☐ Ecological, evolutionary & environmental sciences

For a reference copy of the document with all sections, see nature.com/documents/nr-reporting-summary-flat.pdf

Behavioural & social sciences study design

All studies must disclose on these points even when the disclosure is negative.

Study description	The study is quantitative, involving descriptive analyses and experimental survey data. Study designs are outlined in the Methods section of the paper.
Research sample	The sample for the consumer study was provided by CloudResearch, which recruited participants in the U.S. based on quotas to ensure a nationally representative sample for the U.S. population on three criteria: gender, age and race. CloudResearch was chosen for its higher quality participant pool (relative to MTurk) based on prior analysis and its ability to provide a large sample of consumers of commonly used products/services in the U.S. Further details about the consumer study research sample are provided in Methods, "Consumer experiment design: Setting and sample recruitment" and in Supplementary Information, "Section 2.2: Consumer study results".

The sample for the decision-maker study is from the alumni pool of Executive Education programs at two of our partner organizations. As mentioned in the Methods section, this sample was chosen in order to survey senior managers and leaders who could influence strategic decision-making within their firms. Additionally, partnering with two university programs instead of a specific firm allowed us to access a more diverse sample of companies than prior work that sampled specific types of firms, e.g. innovative firms, startups or small businesses. urther details about the decision-maker study research sample are provided in Methods, "Decision-maker experiment design: Setting and sample recruitment" and in Supplementary Information, "Section 2.3: Decision-maker study results".

Sampling strategy

CloudResearch collected data for our consumer study, ensuring that the sample was nationally representative for the U.S. population based on three criteria: gender, age and race. We performed power calculations to arrive at a rough estimate of the sample size required.

Data for our decision-making study was collected by sending invite emails with the survey link via our partner organizations, who randomly sampled potential study participants from their alumni pool. The sample size we arrived at was based on the maximum sample our partner organizations were comfortable sending emails to.

Data collection

Survey data was collected using Qualtrics survey software. Respondents could complete the survey on their own (without any researchers present at the time of them filling out the survey) using any appropriate web- and browser-enabled device.

Timing

Data for our consumer study was collected between August 29, 2022 and September 7, 2022. An initial data sample for our decision-maker study was collected from November 22, 2022 to December 5, 2022 with a larger sample being collected from December 7, 2022 to December 26, 2022.

Data exclusions

In both experiments, participants were excluded from continuing the survey if they did not provide consent to participate in the study at the beginning of the survey. These included 483 participants in our consumer survey and 8 participants in our decision-maker survey.

Participants in our consumer survey were further restricted from continuing the study if they reported not being a U.S. citizen or being based in the U.S. This excluded 188 participants.

59 participants in the decision-maker study were further excluded if they reported not being employed at the time of the survey.

Finally, participants in both experiments were excluded from analyses if they exhibited inattentiveness during our survey by incorrectly answering specific question(s). This excluded 5609 participants from our consumer study and 66 from our decision-maker study.

These exclusion criteria were pre-established.

Non-participation

In our consumer experiment, 483 participants declined participation by not providing consent to participate at the beginning of the survey. A further 188 participants were dropped out from the study after they reporting not being a U.S. citizen or not being based in the U.S. Finally, 5609 participants were dropped from analyses for incorrectly answering our attention check question.

In our decision-maker study, 8 participants declined participation by not providing consent at the beginning of the survey. 59 participants in the decision-maker study were dropped out if they reported not being employed at the time of the survey. Finally, 66 participants were dropped from analyses for incorrectly answering our attention check questions.

Randomization

Randomization for both survey experiments was completed using Qualtrics survey software.

Reporting for specific materials, systems and methods

We require information from authors about some types of materials, experimental systems and methods used in many studies. Here, indicate whether each material, system or method listed is relevant to your study. If you are not sure if a list item applies to your research, read the appropriate section before selecting a response.

Materials & experimental systems

n/a	Involved in the study
☒	☐ Antibodies
☒	☐ Eukaryotic cell lines
☒	☐ Palaeontology and archaeology
☒	☐ Animals and other organisms
☒	☐ Clinical data
☒	☐ Dual use research of concern
☒	☐ Plants

Methods

n/a	Involved in the study
☒	☐ ChIP-seq
☒	☐ Flow cytometry
☒	☐ MRI-based neuroimaging

Plants

Seed stocks	N/A
Novel plant genotypes	N/A
Authentication	N/A
